# Granulocyte-Macrophage Colony-Stimulating Factor-Activated Neutrophils Express B7-H4 That Correlates with Gastric Cancer Progression and Poor Patient Survival

**DOI:** 10.1155/2021/6613247

**Published:** 2021-03-01

**Authors:** Zhi-guo Shan, Zong-bao Yan, Liu-sheng Peng, Ping Cheng, Yong-sheng Teng, Fang-yuan Mao, Kun Fan, Yuan Zhuang, Yong-liang Zhao

**Affiliations:** ^1^Department of General Surgery and Center of Minimal Invasive Gastrointestinal Surgery, Southwest Hospital, Third Military Medical University, Chongqing 400038, China; ^2^National Engineering Research Center of Immunological Products, Department of Microbiology and Biochemical Pharmacy, College of Pharmacy and Laboratory Medicine, Third Military Medical University, Chongqing 400038, China; ^3^Department of General Surgery, Qijiang Hospital of the First Affiliated Hospital of Chongqing Medical University, Qijiang, Chongqing 401420, China

## Abstract

Neutrophils are prominent components of gastric cancer (GC) tumors and exhibit distinct phenotypes in GC environment. However, the phenotype, regulation, and clinical relevance of neutrophils in human GC are presently unknown. Here, immunohistochemistry, real-time PCR, and flow cytometry analyses were performed to examine levels and phenotype of neutrophils in samples from 41 patients with GC, and also isolated, stimulated, and/or cultured neutrophils for *in vitro* regulation assays. Finally, we performed Kaplan-Meier plots for overall survival by using the log-rank test to evaluate the clinical relevance of neutrophils and their subsets. In our study, neutrophils in tumor tissues were significantly higher than those in nontumor tissues and were positively associated with tumor progression but negatively correlated with GC patient survival. Most intratumoral neutrophils showed an activated CD54^+^ phenotype and expressed high-level immunosuppressive molecule B7-H4. Tumor tissue culture supernatants from GC patients induced neutrophils to express CD54 and B7-H4 in both time-dependent and dose-dependent manners. Locally enriched CD54^+^ neutrophils and B7-H4^+^ neutrophils positively correlated with increased granulocyte-macrophage colony-stimulating factor (GM-CSF) detection *ex vivo*, and *in vitro* GM-CSF induced the expression of CD54 and B7-H4 on neutrophils in a time-dependent and dose-dependent manner. Moreover, GC tumor-derived GM-CSF activated neutrophils and induced neutrophil B7-H4 expression via Janus kinase (JAK)-signal transducer and activator of transcription 3 (STAT3) signaling pathway activation. Furthermore, higher intratumoral B7-H4^+^ neutrophil percentage/number was found in GC patients with advanced tumor node metastasis stage and reduced overall survival following surgery. Our results illuminate a novel regulating mechanism of B7-H4 expression on tumor-activated neutrophils in GC, suggesting that functional inhibition of these novel GM-CSF-B7-H4 pathways may be a suitable therapeutic strategy to treat the immune tolerance feature of GC.

## 1. Introduction

Gastric cancer (GC), with 5-year survival of less than 40%, is one of the leading causes of tumor death in many less-developed countries [1]. Although it is accepted that Helicobacter pylori infection is associated with GC [2, 3], the pathogenesis of GC is presently unknown and its development and prognosis are closely associated with the infiltrating immune cells in the GC environment [4]. Besides tumor cells, different immune cells are infiltrating in the GC environment [5]. Among them, one of the mostly infiltrated immune cells in the GC environment are neutrophils [6]. Currently, many researches focus on the prognosis of peripheral neutrophil number in patients with GC, and it is reported that, in peripheral blood of GC patients, the increased neutrophil/lymphocyte ratio could predict poor survival of GC patients [7]. As to the infiltrating neutrophils in the GC environment, there are studies by using immunohistochemistry showing the relationships between high tumor-infiltrating neutrophils and poor prognosis of GC patients [8]. These studies on peripheral and infiltrating neutrophils together suggest that neutrophils may play pathological roles in GC. However, in humans, virtually nothing is known about the pathological phenotype of neutrophils in GC as well as the underlying regulatory mechanism and clinical relevance of this phenotype of neutrophils in GC. B7-H4, also known as V-set domain containing T cell activation inhibitor 1 (VTCN1), is an immune regulatory protein of the B7 family [9]. It has been reported that B7-H4 negatively regulates T cell immunity [10] and that B7-H4 is regarded as a new checkpoint in human cancers [11]. B7-H4 has been found to be overexpressed in several human cancer types, such as cervical cancer [12] and non-small-cell lung cancer [13], and has also been found to predict patient's survival in human esophageal squamous cell carcinoma [14], renal cell carcinoma [15], and ovarian cancer [16]. As to GC, it has been shown that B7-H4 expression on gastric tumor tissues [17], circulating monocytes [18], or regulatory T cells [19] predicts poor survival of patients suffering from GC. It has also been reported that the Janus kinase (JAK)-signal transducer and activator of transcription 3 (STAT3) signaling pathways are activated in GC cells [20] and GC-associated macrophages [21] by IL-6. However, B7-H4 expression on human primary neutrophils and its regulatory pathway as well as its clinical relevance in GC has not yet been explored.

Herein, we show that neutrophils are highly enriched within the GC environment and that their enrichment positively associated with GC tumor progression but negatively correlated with GC patient survival. Moreover, we demonstrate that GC tumor-derived granulocyte-macrophage colony-stimulating factor (GM-CSF) efficiently activates neutrophils and induces B7-H4 expression on neutrophils by activating the JAK-STAT3 signaling pathways. Furthermore, higher intratumoral B7-H4^+^ neutrophil percentage and higher intratumoral B7-H4^+^ neutrophil number are found in GC patients with advanced tumor node metastasis (TNM) stage and reduced overall survival following surgery.

## 2. Materials and Methods

### 2.1. Patients and Specimens

Fresh gastric tumor, peritumoral, and nontumor (nontumor tissues, at least 5 cm distant from the tumor site) tissues and autologous peripheral blood were obtained from GC patients who underwent surgical resection at the Southwest Hospital of the Third Military Medical University and Qijiang Hospital of the First Affiliated Hospital of Chongqing Medical University. None of these patients had received chemotherapy or radiotherapy before surgery. Patients with infectious diseases, autoimmune disease, or multiprimary cancers were excluded. The clinical stages of tumors were determined according to the TNM classification system of the International Union Against Cancer (8th edition). *Helicobacter pylori* (*H. pylori*) infection was determined by a serology test for specific anti-*H. pylori* antibodies. Antibodies and other reagents are listed in Supplementary Table 1. The biopsy specimens were obtained under protocols approved by the ethics committees of Southwest Hospital of the Third Military Medical University and Qijiang Hospital of the First Affiliated Hospital of Chongqing Medical University, and an informed consent was obtained from all patients. We confirm that all methods were performed in accordance with the relevant guidelines and regulations. The clinical characteristics of patients with GC were presented in Supplementary Table 2.

### 2.2. Immunohistochemistry

According to our previously established methods [21], paraformaldehyde-fixed and paraffin-embedded samples were cut into 5 *μ*m sections. For immunohistochemical staining, the sections were incubated with rabbit anti-human myeloperoxidase (MPO) and then were stained by horseradish peroxidase (HRP) anti-rabbit immunoglobulin G (IgG) followed by diaminobenzidine. All the sections were finally counterstained with hematoxylin and examined using a microscope (Nikon Eclipse 80i; Nikon). All sections were analyzed independently by two experienced pathologists who did not have access to the clinical data of patients. Five fields were observed in each section, and the cells with uniform brown granules were counted at 200x magnification in each case using average values.

### 2.3. Isolation of Single Cells from Tissues of GC Patients

According to our previously established methods [6], fresh tissues were washed 3 times with Hank's solution containing 1% fetal calf serum before being cut into small pieces. The specimens were then collected in RPMI 1640 medium containing collagenase IV (1 mg/ml) and deoxyribonuclease I (10 mg/ml) and mechanically dissociated using the gentle MACS Dissociator (Miltenyi Biotec). Dissociated cell suspensions were further incubated for 1 h at 37°C under a continuous rotation. The cell suspensions were then filtered through a 70 *μ*m cell strainer (BD Labware). Cell viability, as determined by trypan blue exclusion staining, was typically >90%.

### 2.4. Isolation of Neutrophils

According to our previously established methods [6], peripheral blood mononuclear cells (PBMCs) from healthy donors and GC patients were isolated by density gradient centrifugation using Ficoll-Paque Plus. Blood neutrophils were harvested after lysis of red blood cells with lysis solution from non-PBMCs. The cells were used unless their viability was determined >90% and their purity was determined >95%.

### 2.5. Preparation of TTCS and NTCS and Supernatant-Conditioned Neutrophils

According to our previously established methods [6], tumor tissue culture supernatants (TTCS) or nontumor tissue culture supernatants (NTCS) were prepared by plating autologous tumor or nontumor gastric tissues in 1 ml RPMI 1640 medium for 24 h. The supernatant was then centrifuged and harvested. To generate supernatant-conditioned neutrophils, neutrophils from healthy doors were first harvested and cultured with 50% TTCS or 50% NTCS for 12 h, and then washed with RPMI-1640 for 3 times. Neutrophils cultured with RPMI-1640 medium were used as controls.

### 2.6. Neutrophil Stimulation

Neutrophils from healthy donors were stimulated with 50% TTCS or 50% autologous NTCS, or 50% TTCS with a neutralizing antibody against human GM-CSF (10 *μ*g/ml), or 50% autologous NTCS with human recombinant (hr) GM-CSF (100 ng/ml) for 12 h, or were stimulated with TTCS (10%, 20%, or 50%) or hr GM-CSF (25, 50, or 100 ng/ml) for 12 h, or were stimulated with 50% TTCS or hr GM-CSF (100 ng/ml) for 3, 6, or 12 h. In some cases, blocking antibody for GM-CSF receptor (GM-CSFR) (20 *μ*g/ml) was added into neutrophil suspensions and incubated for 2 h before stimulation. After stimulation, the cells were harvested for flow cytometric analysis and western blot. For the signaling pathway inhibition experiments, these neutrophils were pretreated with AG490 (a JAK inhibitor), FLLL32 (an STAT3 inhibitor), BAY 11-7082 (an I*κ*B*α* inhibitor), SP600125 (a JNK inhibitor), SB203580 (a MAPK inhibitor), U0126 (an MEK-1 and MEK-2 inhibitor), Wortmannin (a PI3K inhibitor), or GSK-3*β* inhibitor (5 *μ*l, 20 *μ*M) for 1 h; then, the cells were stimulated with 50% TTCS or hr GM-CSF (100 ng/ml) for 12 h and harvested as above. Since the inhibitor was dissolved in DMSO, parallel cell groups were treated with DMSO (5 *μ*l) or culture media as controls.

### 2.7. Flow Cytometry

According to our previously established methods [6], cell surface markers were stained with specific or isotype control antibodies. Flow cytometric analysis was performed according to standard protocols. The cells were analyzed by multicolor flow cytometry with FACSCanto™ (BD Biosciences). Data were analyzed with FlowJo software (TreeStar) or FACSDiva software (BD Biosciences).

### 2.8. Real-Time PCR

According to our previously established methods [6], RNA of biopsy specimens was extracted with TRIzol reagent. The RNA samples were reversed transcribed into cDNA with PrimeScript™ RT reagent kit. Real-time PCR was performed on an IQ5 (Bio-Rad) with real-time PCR Master Mix according to the manufacturer's specifications. The mRNA expression of MPO gene was measured using the SYBR green method with the relevant primers (Supplementary Table 3). For human samples, human GAPDH mRNA level served as a normalizer, and its level in nontumor tissues served as a calibrator. The relative gene expression was expressed as fold change of relevant mRNA calculated by the *^ΔΔ^*Ct method.

### 2.9. Western Blotting

According to our previously established methods [6], western blot assays were performed on 10%-15% SDS-PAGE gel transferred PVDF membranes using equivalent amounts of cell lysate protein for each sample. Five percent skimmed milk or three percent BSA was used for blocking the PVDF membranes. Human STAT3 and p-STAT3 were detected with anti-STAT3 and anti-p-STAT3 antibodies, respectively. This was followed by incubation with HRP-conjugated secondary antibodies. Bound proteins were visualized by using SuperSignal® West Dura Extended Duration Substrate kit.

### 2.10. Enzyme-Linked Immunosorbent Assay (ELISA)

According to our previously established methods [6], human gastric tissues from specimens were collected, homogenized in 1 ml sterile protein extraction reagent, and centrifuged. Tissue supernatants were collected for ELISA. Concentrations of GM-CSF in the tissue supernatants or in the TTCS and NTCS from autologous tumor or nontumor gastric tissues were determined using ELISA kits according to the manufacturer's instructions.

### 2.11. Microarray Experiments

Gene expression profiles of human tumor tissues from GC patients were analyzed with the Affymetrix GeneChip Human Gene 1.0 ST Array (Affymetrix), strictly following the manufacturer's protocol. Microarray experiments were performed at the Genminix Informatics (China) with the microarray service certified by Affymetrix.

### 2.12. Statistical Analysis

Results are expressed as the mean ± SEM. The Student *t*-test was generally used to analyze the differences between two groups, but when the variances differed, the Mann–Whitney *U* test was used. For multigroup data analysis, an ANOVA analysis was used. Correlations between parameters were assessed using the Pearson correlation analysis and linear regression analysis as appropriate. Overall survival was defined as the interval between surgery and death. The known tumor-unrelated deaths (e.g., accidental death) were excluded from the death record for this study. Cumulative survival time was calculated by the Kaplan-Meier method, and survival was measured in months; the log-rank test was applied to compare between 2 groups. SPSS Statistical Software (version 13.0) was used for all statistical analyses. All data were analyzed using 2-tailed tests, and *P* < 0.05 was considered statistically significant.

## 3. Results

### 3.1. Neutrophils Are Enriched in Human GC Environment with Tumor Progression and Associated with Poor Patient Survival

**Figure 1:**
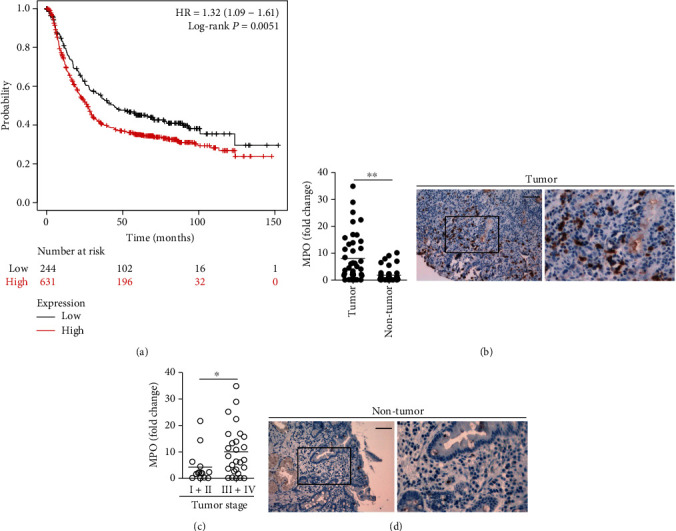
Neutrophils are enriched in human GC environment with tumor progression and associated with poor patient survival. (a) Kaplan-Meier plots for intratumoral MPO expression in GC patients (overall survival in Kaplan-Meier plotter website). (b) Expression of MPO between autologous tumor and nontumor tissues (*n* = 41) was analyzed. (c) Expression of MPO in GC tumors among TNM stages was compared. Human GAPDH mRNA level served as a normalizer, and its level in nontumor tissues served as a calibrator. The relative gene expression was expressed as fold change of relevant mRNA calculated by the *^ΔΔ^*Ct method. (d) Representative analysis of MPO^+^ (brown) neutrophil distributions in tumor and nontumor tissues of GC patients by immunohistochemical staining. Scale bars: 100 microns. MPO: myeloperoxidase. The horizontal bars in (b) and (c) represent mean values. Each ring or dot in (b) and (c) represents 1 patient. ^∗^*P* < 0.05, ^∗∗^*P* < 0.01, and ^n.s.^*P* > 0.05 for groups connected by horizontal lines.

Kaplan-Meier survival curves were obtained in the KM plots database to characterize the correlation of myeloperoxidase (MPO), a marker for neutrophils that has been reported in many human cancers and diseases [22–27], with GC prognosis, which showed that a higher level of MPO in GC tumors associated with poorer overall survival of patients with GC (Figure 1(a)). Notably, MPO expression was significantly higher in tumor tissues than that in nontumor tissues (Figure 1(b)). Moreover, as the cancer progressed, we found that the expression of MPO significantly increased in the tested tumor samples (Figure 1(c)). In keeping with this finding, an increased MPO expression was correlated with increased tumor size and advanced lymphatic invasion (Supplementary Figure 1). Furthermore, immunohistochemical staining also showed that MPO^+^ neutrophils were accumulated in tumors compared to nontumor tissues (Figure 1(d)). Taken together, these findings suggest that neutrophils are increased in GC tumors and that intratumoral neutrophils are associated with tumor progression and poor survival for GC patients.

### 3.2. B7-H4 Expression and Activation of Neutrophils Are Correlated in Human GC Environment

**Figure 2:**
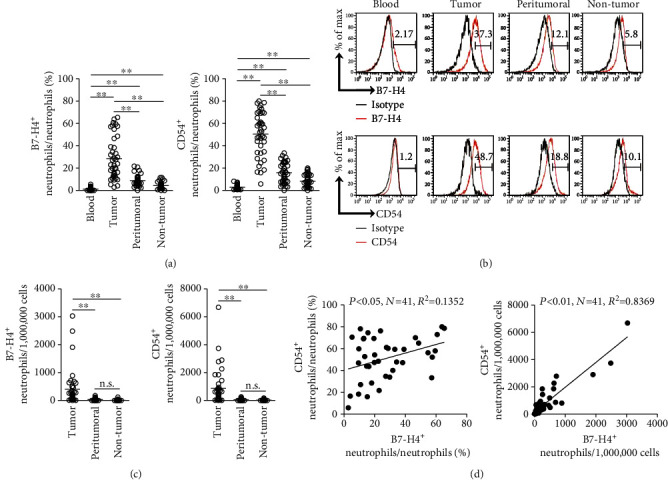
B7-H4 expression and activation of neutrophils are correlated in human GC environment. (a) Statistics analysis of the percentage of CD54^+^ neutrophils and B7-H4^+^ neutrophils in total neutrophils in each sample of patients with GC (*n* = 41). (b) Expression of molecules CD54 and B7-H4 on neutrophils. Color histograms represent staining of neutrophil activation marker CD54 and immunosuppressive molecule B7-H4; black: isotype control. (c) Statistics analysis of the number of CD54^+^ neutrophils and B7-H4^+^ neutrophils per million total cells in each sample of patients with GC (*n* = 41). (d) The correlations between CD54^+^ neutrophils and B7-H4^+^ neutrophils in GC tumors were analyzed. Results are expressed as the percentage of CD54^+^ neutrophils and B7-H4^+^ neutrophils in neutrophils or the number of CD54^+^ neutrophils and B7-H4^+^ neutrophils per million total cells in tumor tissues of patients with GC. The horizontal bars in (a) and (c) represent mean values. Each ring or dot in (a), (c), and (d) represents 1 patient. ^∗^*P* < 0.05, ^∗∗^*P* < 0.01, and ^n.s.^*P* > 0.05 for groups connected by horizontal lines.

We next analyzed the immunophenotyping of these enriched intratumoral neutrophils. First, we found that peripheral neutrophils from GC patients expressed little neutrophil activation marker CD54 (Figures 2(a)–2(c)). Notably, intratumoral neutrophils expressed a significantly higher level of CD54 than those expressed on peritumoral and nontumor tissue neutrophils (Figures 2(a)–2(c)), suggesting an activation of neutrophils in the GC environment. Interestingly, intratumoral neutrophils from GC patients expressed a significantly higher level of immunosuppressive molecule B7-H4 than those expressed on peritumoral and nontumor tissue neutrophils (Figures 2(a)–2(c)); however, peripheral neutrophils expressed little B7-H4 (Figures 2(a)–2(c)). Moreover, significant correlations were found between the levels of CD54 and B7-H4 expression on neutrophils in GC tumors analyzed (Figure 2(d)). We also detected other neutrophil activation markers such as CD11b and CD16 [28] on peripheral, intratumoral, peritumoral, and nontumor tissue neutrophils, and found that intratumoral neutrophils from GC patients expressed a significantly higher level of CD11b and CD16 than those expressed on peritumoral and nontumor tissue neutrophils; however, peripheral neutrophils expressed little CD11b and CD16 (Supplementary Figure 2). Taken together, the above data indicate that tumor-infiltrating neutrophils exhibit an activated and highly B7-H4-expressing phenotype.

### 3.3. Human GC Environments Maintain Neutrophil-Activated and Highly B7-H4-Expressing Phenotype

**Figure 3:**
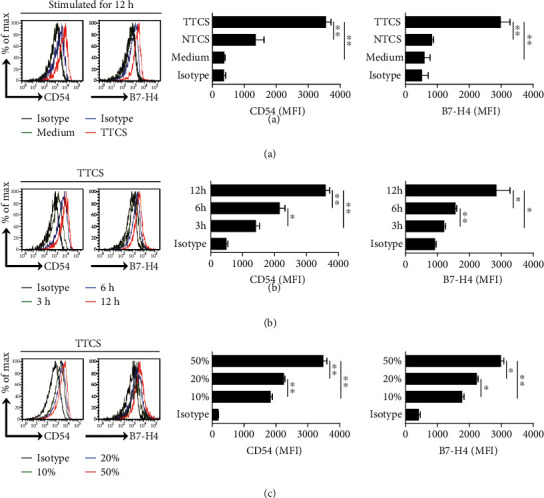
Human GC environments maintain neutrophil-activated and highly B7-H4-expressing phenotype. (a) Expressions of CD54 and B7-H4 on neutrophils exposed to 50% TTCS and 50% NTCS from autologous GC patients, or to medium control for 12 h. Black: isotype control. MFI: mean fluorescence intensity. (b) Expressions of CD54 and B7-H4 on neutrophils exposed to 50% TTCS from GC patients for 3, 6, or 12 h. Black: isotype control. MFI: mean fluorescence intensity. (c) Expressions of CD54 and B7-H4 on neutrophils exposed to 10%, 20%, or 50% TTCS from GC patients for 12 h. Black: isotype control. MFI: mean fluorescence intensity. ^∗^*P* < 0.05 and ^∗∗^*P* < 0.01 for groups connected by horizontal lines.

Furthermore, we next hypothesized that GC environments contribute to the activated and highly B7-H4-expressing phenotype of neutrophils. Consistent with our hypothesis, we stimulated neutrophils with NTCS or TTCS from autologous tumor or nontumor gastric tissues, and found that, compared to NTCS-conditioned neutrophils, TTCS-conditioned neutrophils significantly upregulated CD54 and B7-H4 expression (Figure 3(a)). We also found that TTCS-conditioned neutrophils upregulated CD54 and B7-H4 expression in a time-dependent manner (Figure 3(b)) as well as in a dose-dependent manner (Figure 3(c)). These findings together imply that GC environment is involved in the activation and B7-H4 expression on neutrophils.

### 3.4. GM-CSF Activates Neutrophils and Induces B7-H4 Expression on Neutrophils

**Figure 4:**
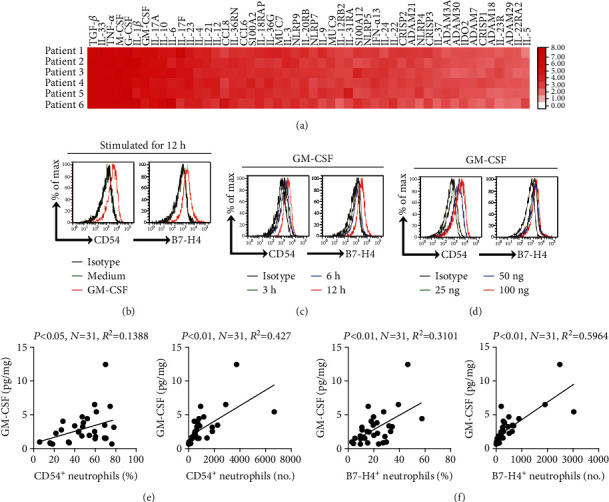
GM-CSF activates neutrophils and induces B7-H4 expression on neutrophils. (a) Clustering of microarray data for the expression of 50 proinflammatory cytokine genes in human tumor tissues from 6 GC patients. (b) Expressions of CD54 and B7-H4 on neutrophils exposed to GM-CSF (100 ng/ml), or to medium control for 12 h. Black: isotype control. (c) Expressions of CD54 and B7-H4 on neutrophils exposed to GM-CSF (100 ng/ml) for 3, 6, or 12 h. Black: isotype control. (d) Expressions of CD54 and B7-H4 on neutrophils exposed to GM-CSF (25, 50, or 100 ng/ml) for 12 h. Black, isotype control. (e) The correlations between GM-CSF and CD54^+^ neutrophils in GC tumors were analyzed. Results are expressed as the percentage of CD54^+^ neutrophils in neutrophils or the number of CD54^+^ neutrophils per million total cells and GM-CSF concentration in GC tumor tissues. (f) The correlations between GM-CSF and B7-H4^+^ neutrophils in GC tumors were analyzed. Results are expressed as the percentage of B7-H4^+^ neutrophils in neutrophils or the number of B7-H4^+^ neutrophils per million total cells and GM-CSF concentration in GC tumor tissues. Each dot in (e) and (f) represents 1 patient.

Tumor microenvironment can possess various soluble inflammatory factors, including cytokines with potential proinflammatory effects. To see which cytokines might activate neutrophils and induce B7-H4 expression on neutrophils, we first screened proinflammatory cytokines in human GC environments by a microarray (Figure 4(a)) and stimulated normal neutrophils with highly expressed cytokines including TGF-*β*, IL-33, TNF-*α*, M-CSF, G-CSF, IL-1*β*, GM-CSF, IL-17A, IL-10, IL-6, IL-17F, IL-23, IL-4, IL-21, and IL-12. We found that only GM-CSF remarkably upregulated the expression of B7-H4 on neutrophils (Figure 4(b) and Supplementary Figure 3). We also found that GM-CSF upregulated B7-H4 expression on neutrophils in a time-dependent manner (Figure 4(c)) as well as in a dose-dependent manner (Figure 4(d)). Similar observations were made when analyzing the induction of neutrophil activation marker CD54 on neutrophils by GM-CSF (Figures 4(b)–4(d)). Moreover, significant correlations were found between the concentrations of GM-CSF and the levels of CD54^+^ neutrophils (Figure 4(e)) as well as between the concentrations of GM-CSF and the levels of B7-H4^+^ neutrophils (Figure 4(f)) in GC tumors analyzed. Taken together, the above data indicate that GM-CSF activates neutrophils and induces B7-H4 expression on neutrophils.

### 3.5. Tumor-Derived GM-CSF Activates Neutrophils and Induces B7-H4 Expression on Neutrophils via Activating the JAK-STAT3 Pathway

**Figure 5:**
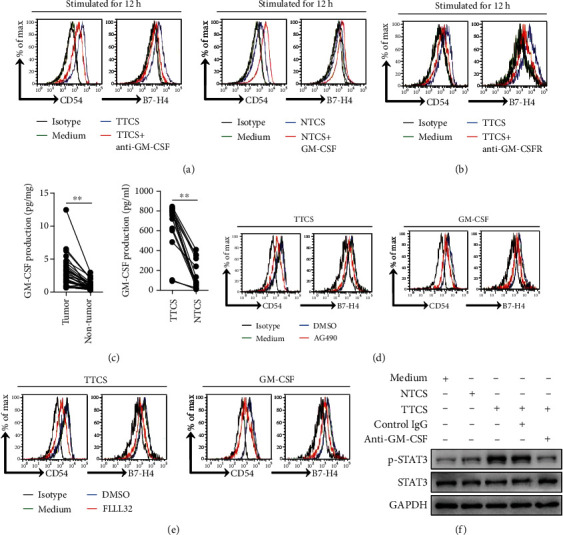
Tumor-derived GM-CSF activates neutrophils and induces B7-H4 expression on neutrophils via activating the JAK-STAT3 pathway. (a) Expressions of CD54 and B7-H4 on neutrophils exposed to 50% TTCS with anti-GM-CSF antibody or 50% NTCS with GM-CSF for 12 h. Black: isotype control. (b) Expressions of CD54 and B7-H4 on neutrophils exposed to 50% TTCS pretreated with anti-GM-CSF receptor (GM-CSFR) antibody for 12 h. Black: isotype control. (c) GM-CSF concentration between autologous tumor and nontumor tissues (*n* = 31) or between autologous TTCS and NTCS (*n* = 14) was analyzed. (d) Expressions of CD54 and B7-H4 on neutrophils exposed to 50% TTCS or GM-CSF with or without JAK signal transduction inhibitor AG490 for 12 h. (e) Expressions of CD54 and B7-H4 on neutrophils exposed to 50% TTCS or GM-CSF with or without STAT3 phosphorylation inhibitor FLLL32 for 12 h. (f) STAT3 and p-STAT3 in neutrophils exposed to 50% TTCS and 50% NTCS from autologous GC patients, or to medium control, or to 50% TTCS with anti-GM-CSF antibody or control IgG were analyzed by western blot. Each dot in (b) represents 1 patient. ^∗^*P* < 0.05 for groups connected by horizontal lines.

Next, to evaluate the potential role of GC tumor-derived GM-CSF in B7-H4 induction on neutrophils, we added neutralizing antibody against GM-CSF into TTCS/neutrophil coculture. Interestingly, antibody blockade of GM-CSF efficiently inhibited the induction of B7-H4 on neutrophils (Figure 5(a)). Consistent with these findings, provision of exogenous GM-CSF into NTCS/neutrophil coculture significantly increased the B7-H4 expression on neutrophils (Figure 5(a)). Similar observations were made when analyzing the induction of neutrophil activation marker CD54 (Figure 5(a)). Furthermore, blocking the GM-CSF receptor (GM-CSFR) inhibited the induction of B7-H4 and CD54 on TTCS-stimulated neutrophils (Figure 5(b)). Next, we found that the concentrations of GM-CSF in tumor tissues or TTSC were significantly increased when compared to those in nontumor tissues or NTCS (Figure 5(c)). These findings show that GC tumor-derived GM-CSF plays an essential role in neutrophil activation and B7-H4 induction.

To see which signaling pathways might operate in neutrophil activation and B7-H4 induction, we first pretreated neutrophils with corresponding inhibitors and then exposed them to the indicated TTCS. The results showed that only blocking the signal transduction of JAK with inhibitor AG490 and/or abolishing the phosphorylation of STAT3 with inhibitor FLLL32 effectively suppressed B7-H4 expression on TTCS-conditioned neutrophils (Figures 5(d) and 5(e) and Supplementary Figure 4). Similar observations were made when analyzing the induction of neutrophil activation marker CD54 (Figures 5(d) and 5(e)) or when analyzing GM-CSF-induced CD54 and B7-H4 on neutrophils (Figures 5(d) and 5(e)). Furthermore, STAT3, a direct JAK-STAT3 pathway downstream substrate, was predominantly phosphorylated in neutrophils after treatment with TTCS, and this phosphorylation was abolished when blocking GM-CSF (Figure 5(f)). Taken together, these data indicate that the activation of the JAK-STAT3 signaling pathway is crucial for neutrophil activation and B7-H4 induction by GM-CSF in the GC environment.

### 3.6. B7-H4^+^ Neutrophils Correlate with Advanced Tumor Stage and Poor Survival in Patients with GC

**Figure 6:**
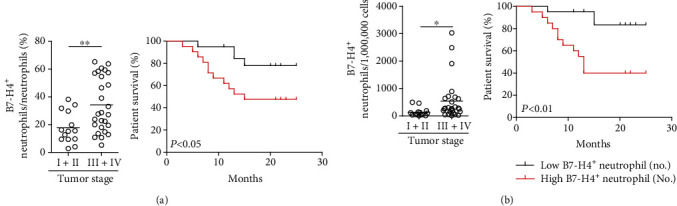
B7-H4^+^ neutrophils correlate with advanced tumor stage and poor survival in patients with GC. (a) Intratumoral B7-H4^+^ neutrophil percentage among TNM stages was compared. Kaplan-Meier plots for overall survival of GC patients by median intratumoral B7-H4^+^ neutrophil percentage (22.9%). (b) Intratumoral B7-H4^+^ neutrophil number among TNM stages was compared. Kaplan-Meier plots for overall survival of GC patients by median intratumoral B7-H4^+^ neutrophil number (196 per million). The horizontal bars in (a) and (b) represent mean values. Each ring in (a) and (b) represents 1 patient. ^∗^*P* < 0.05, ^∗∗^*P* < 0.01, and ^n.s.^*P* > 0.05 for groups connected by horizontal lines.

Finally, we studied whether increased B7-H4^+^ neutrophils were associated with tumor stage and GC prognosis. We found that intratumoral B7-H4^+^ neutrophil percentage in patients with advanced GC was significantly higher than that in patients with early GC (Figure 6(a)), suggesting that B7-H4^+^ neutrophils accumulate at tumor site during tumor progression. We also evaluated the prognostic value of intratumoral B7-H4^+^ neutrophil percentage on the overall survival of GC patients. Comparing patients with high (≥22.9% median level) versus low (<22.9% median level) B7-H4^+^ neutrophil percentage level, the 25-month survival rate was significantly lower for those within the higher B7-H4^+^ neutrophil percentage group (Figure 6(a)). Similar results were obtained when the patient cohort was stratified based on intratumoral B7-H4^+^ neutrophil number (Figure 6(b)). In keeping with this finding, an increased B7-H4^+^ neutrophil was correlated with increased tumor size and advanced tumor stage (Supplementary Figure 5). Taken together, these findings indicate that increased intratumoral B7-H4^+^ neutrophils are associated with tumor progression and poor survival for GC patients.

## 4. Discussion

In this study, we have shown that, within the GC environment, neutrophils with CD54^+^-activated and highly B7-H4-expressing phenotype significantly increase with tumor progression. Although increased neutrophils have already been described in patients with many types of tumors including GC [20], to our knowledge, this is the first demonstration of a statistically significant correlation between prevalently high B7-H4-expressing neutrophils in human tumors and poor prognosis; it is also the first demonstration for tumor-derived GM-CSF to induce B7-H4 on neutrophils within the tumor environment.

Neutrophils have been found to be enriched in tumors; however, very little is currently known about the phenotype of tumor-infiltrating neutrophils, as well as its regulation and clinical relevance. In human GC, it has been reported that increased neutrophils in peripheral blood [7] and tumor tissues [8] could predict poor prognosis of patients with GC. Upon these previous observations, we now have significantly expanded the profiling of tumor-infiltrating neutrophils that within GC, they are phenotypically distinct from their peripheral counterparts. Firstly, we confirmed that tumor-infiltrating neutrophils exhibited an activated phenotype characterized by the increase of molecule CD54, compared with peripheral cohorts [21]. Most interestingly, we further demonstrate these activated neutrophils expressed high level molecule B7-H4, an important immune checkpoint member of the B7-CD28 family, indicating that the main role of tumor-infiltrating neutrophils is likely to be modulating immune function.

It has been known that immune suppression exhibits a hallmark of cancer [22]. It is shown that B7-H4-mediated immune suppression in antitumor immunity is one of the main mechanisms contributing to the dysfunction of T cells [23]. However, another research has identified a promoting role of antitumor immunity by B7-H4 [24]. The difference on the opposite role of B7-H4 may own to the B7-H4 expression cell type. B7-H4 expression by nonhematopoietic cells such as fibroblasts in the tumor microenvironment promotes antitumor immunity in a mouse model of mammary tumorigenesis [24]; however, B7-H4^+^ tumor macrophages exert suppressive functions in human ovarian carcinoma [25]. Our results are consistent with the latter study showing B7-H4 expression on another cell type of hematopoietic cells in GC, and we are the first to report the high expression of B7-H4 on tumor-infiltrating neutrophils in human GC tumors, which may emphasize the importance of B7-H4-associated pathway in tumor-related immune suppression. We also detected other immune checkpoint receptors such as CTLA-4 and PD-1, and found that there were no significances of expressions of CTLA-4 and PD-1 among intratumoral, peritumoral, and nontumor tissue neutrophils (data not shown), maybe suggesting a relative specificity of B7-H4 induction in neutrophils in GC.

B7-H4 upregulation occurs during inflammation in the tumor environment. It has been shown that IL-6 effectively induces the upregulation of B7-H4 on human macrophages in ovarian carcinoma [25]. Our results are consistent with the study showing cytokine-inducing effect on neutrophils' B7-H4 expression in GC. GM-CSF, as a proinflammatory and pluripotent cytokine, is reported to regulate hemopoiesis as well as immune response [26]. The GM-CSF-secreting tumors, including lung tumor [27] and colorectal tumor [28], are one of the most rapidly advancing tumors with multiple proinflammatory cytokines in the tumor environment [29]. Here, we show a higher production of GM-CSF in tumors than that in nontumor tissues and positive correlations between GM-CSF production and CD54^+^ or B7-H4^+^ neutrophils within the GC environment. Importantly, we further identify GM-CSF as a novel proinflammatory factor to induce B7-H4 on neutrophils in GC and show that GC tumor-derived GM-CSF effectively activates the JAK-STAT3 pathway to induce this B7-H4 expression on tumor-infiltrating neutrophils.

Cell stimulation by cytokines or growth factors induces JAK activation, resulting STAT3 phosphorylation, and phosphorylated STAT3 directly mediate signaling from the cell membrane to the nucleus [30]. Many cytokines can activate the JAK-STAT3 signaling pathway. It has been shown that IL-6-induced JAK-STAT3 signaling pathway activation plays important roles in endothelial cell activation [31] that is mainly mediated by IL-6-induced Tyr705 phosphorylation in STAT3 [32], which resembles our data on B7-H4 regulation by GM-CSF-induced Tyr705 phosphorylation of STAT3 in neutrophils. As increasing evidence including our previous studies indicates that PD-L1, another immune inhibitory molecule, is induced by GM-CSF to express on neutrophils in GC [6] or on myeloid-derived suppressor cells in live metastases [33] via activating the JAK-STAT3 signaling pathway, we have now added B7-H4 onto that list as it is also induced to express on tumor-infiltrating neutrophils that responded to GC-derived GM-CSF. It has also been reported that GM-CSF could upregulate the expression of CD54 on mouse neutrophils sorted from the bone marrow in acute peritonitis model [34], which resembles our data on CD54 regulation on tumor-infiltrating neutrophils by GM-CSF in GC. Additionally, our results indicate that Tyr705 phosphorylation of STAT3 plays an important role in this induction of CD54 on tumor-infiltrating neutrophils by GM-CSF in the GC environment and provides evidence that tumor-infiltrating neutrophils increase expression of both CD54 and B7-H4, which appear to be in both GM-CSF-dose-dependent and time-dependent manners.

Importantly, our findings also shed light on the clinical relevance of B7-H4^+^ neutrophils in GC. Specifically, we have shown that increased frequencies and numbers of intratumoral B7-H4^+^ neutrophils predict lower rates of GC patient survival. As the relationship between B7-H4 expression and tumor vasculature in renal cell carcinoma and their associations with advanced cancer progression and poor survival [15], intratumoral B7-H4^+^ neutrophil frequencies or numbers might prove useful clinical markers for GC.

Collectively, based on our *in vitro* and *ex vivo* data, we identify a novel pathway involving the activation of neutrophils and the induction of B7-H4 expression on neutrophils within GC. First, within the GC environment, GM-CSF with proinflammatory feature is produced. Second, released GM-CSF has effects on intratumoral neutrophils by activating the JAK-STAT3 signaling pathway. Third, the GM-CSF-activated JAK-STAT3 signaling pathway mediates intratumoral neutrophil activation with increasing CD54 expression, a process that is accompanied by the induction of B7-H4 expression on these neutrophils. In this way, neutrophils appear to acquire CD54^+^B7-H4^+^ phenotype via the GM-CSF-JAK-STAT3 pathway, which is consistent with our observations that advanced tumor staging and poor patient prognosis is associated with a significant increase of B7-H4^+^ neutrophils in GC tumors. In the future, therapeutics is aimed at interfering these tumor-infiltrating neutrophils and their regulatory pathway may be developed to provide novel strategies for GC treatment.

## 5. Conclusions

Our results illuminate a novel regulating mechanism of B7-H4 expression on tumor-activated neutrophils in GC, suggesting that functional inhibition of these novel GM-CSF-B7-H4 pathways may be a suitable therapeutic strategy to treat the immune tolerance feature of GC.

## Data Availability

The data that support the findings of this study are available from the corresponding author upon reasonable request.

## Abbreviations

GC: Gastric cancer
PBMCs: Peripheral blood mononuclear cells
IL: Interleukin
GM-CSF: Granulocyte-macrophage colony-stimulating factor
JAK: Janus kinase
STAT3: Signal transducer and activator of transcription 3
MPO: Myeloperoxidase
TNM: Tumor node metastasis
TTCS: Tumor tissue culture supernatants
NTCS: Nontumor tissue culture supernatants.

## Supplementary Materials

Supplementary 1 Supplementary Figure 1: MPO expression and its potential correlations with clinical parameters. MPO expression in GC tumors was analyzed for correlations with clinical pathological parameters. ^∗^*P* < 0.05, ^∗∗^*P* < 0.01, and ^n.s.^*P* > 0.05 for groups connected by horizontal lines. Each dot represents 1 patient. CEA: carcinoembryonic antigen; *H. pylori* Ab: *Helicobacter pylori* antibody. Supplementary Figure 2: statistics analysis of expressions of CD11b and CD16 on neutrophils in each sample of patients with GC (*n* = 41). The horizontal bars in panels represent mean values. Each ring in panels represents 1 patient. ^∗^*P* < 0.05, ^∗∗^*P* < 0.01, and ^n.s.^*P* > 0.05 for groups connected by horizontal lines. Supplementary Figure 3: expression of B7-H4 on neutrophils exposed to G-CSF, M-CSF, TNF-*α*, TGF-*β*, IL-1*β*, IL-4, IL-6, IL-10, IL-12, IL-17A, IL-17F, IL-21, IL-23, or IL-33 (100 ng/ml) for 12 h. Black: isotype control. Supplementary Figure 4: expression of B7-H4 on neutrophils exposed to 50% TTCS with or without BAY 11-7082 (an I*κ*B*α* inhibitor), U0126 (an MEK-1 and MEK-2 inhibitor), SP600125 (a JNK inhibitor), SB203580 (a MAPK inhibitor), Wortmannin (a PI3K inhibitor), or GSK-3*β* inhibitor for 12 h. Black: isotype control. Supplementary Figure 5: intratumoral B7-H4^+^ neutrophil percentage or number and its potential correlations with clinical parameters. Intratumoral B7-H4^+^ neutrophil percentage (a) or intratumoral B7-H4^+^ neutrophil number (b) in GC tumors was analyzed for correlations with clinical pathological parameters. ^∗^*P* < 0.05, ^∗∗^*P* < 0.01, and ^n.s.^*P* > 0.05 for groups connected by horizontal lines. Each dot represents 1 patient. CEA: carcinoembryonic antigen; *H. pylori* Ab: *Helicobacter pylori* antibody.

Supplementary 2 Supplementary Table 1: antibodies and other reagents. Supplementary Table 2: clinical characteristics of 41 patients with gastric cancer. Supplementary Table 3: primer sequences for real-time PCR analysis.
