# Supplementary material for: Granulocyte-Macrophage Colony-Stimulating Factor-Activated Neutrophils Express B7-H4 That Correlates with Gastric Cancer Progression and Poor Patient Survival
Source: J Immunol Res. 2021 Mar 1;2021:6613247. doi: 10.1155/2021/6613247 (PMC7962878; doi:10.1155/2021/6613247)
Supplement: Supplementary 1 — Supplementary Figure 1: MPO expression and its potential correlations with clinical parameters. MPO expression in GC tumors was analyzed for correlations with clinical pathological parameters. ∗P < 0.05, ∗∗P < 0.01, and n.s.P > 0.05 for groups connected by horizontal lines. Each dot represents 1 patient. CEA: carcinoembryonic antigen; H. pylori Ab: Helicobacter pylori antibody. Supplementary Figure 2: statistics analysis of expressions of CD11b and CD16 on neutrophils in each sample of patients with GC (n = 41). The horizontal bars in panels represent mean values. Each ring in panels represents 1 patient. ∗P < 0.05, ∗∗P < 0.01, and n.s.P > 0.05 for groups connected by horizontal lines. Supplementary Figure 3: expression of B7-H4 on neutrophils exposed to G-CSF, M-CSF, TNF-α, TGF-β, IL-1β, IL-4, IL-6, IL-10, IL-12, IL-17A, IL-17F, IL-21, IL-23, or IL-33 (100 ng/ml) for 12 h. Black: isotype control. Supplementary Figure 4: expression of B7-H4 on neutrophils exposed to 50% TTCS with or without BAY 11-7082 (an IκBα inhibitor), U0126 (an MEK-1 and MEK-2 inhibitor), SP600125 (a JNK inhibitor), SB203580 (a MAPK inhibitor), Wortmannin (a PI3K inhibitor), or GSK-3β inhibitor for 12 h. Black: isotype control. Supplementary Figure 5: intratumoral B7-H4+ neutrophil percentage or number and its potential correlations with clinical parameters. Intratumoral B7-H4+ neutrophil percentage (a) or intratumoral B7-H4+ neutrophil number (b) in GC tumors was analyzed for correlations with clinical pathological parameters. ∗P < 0.05, ∗∗P < 0.01, and n.s.P > 0.05 for groups connected by horizontal lines. Each dot represents 1 patient. CEA: carcinoembryonic antigen; H. pylori Ab: Helicobacter pylori antibody. [file 6613247.f1.doc]

**Supplementary Figures**


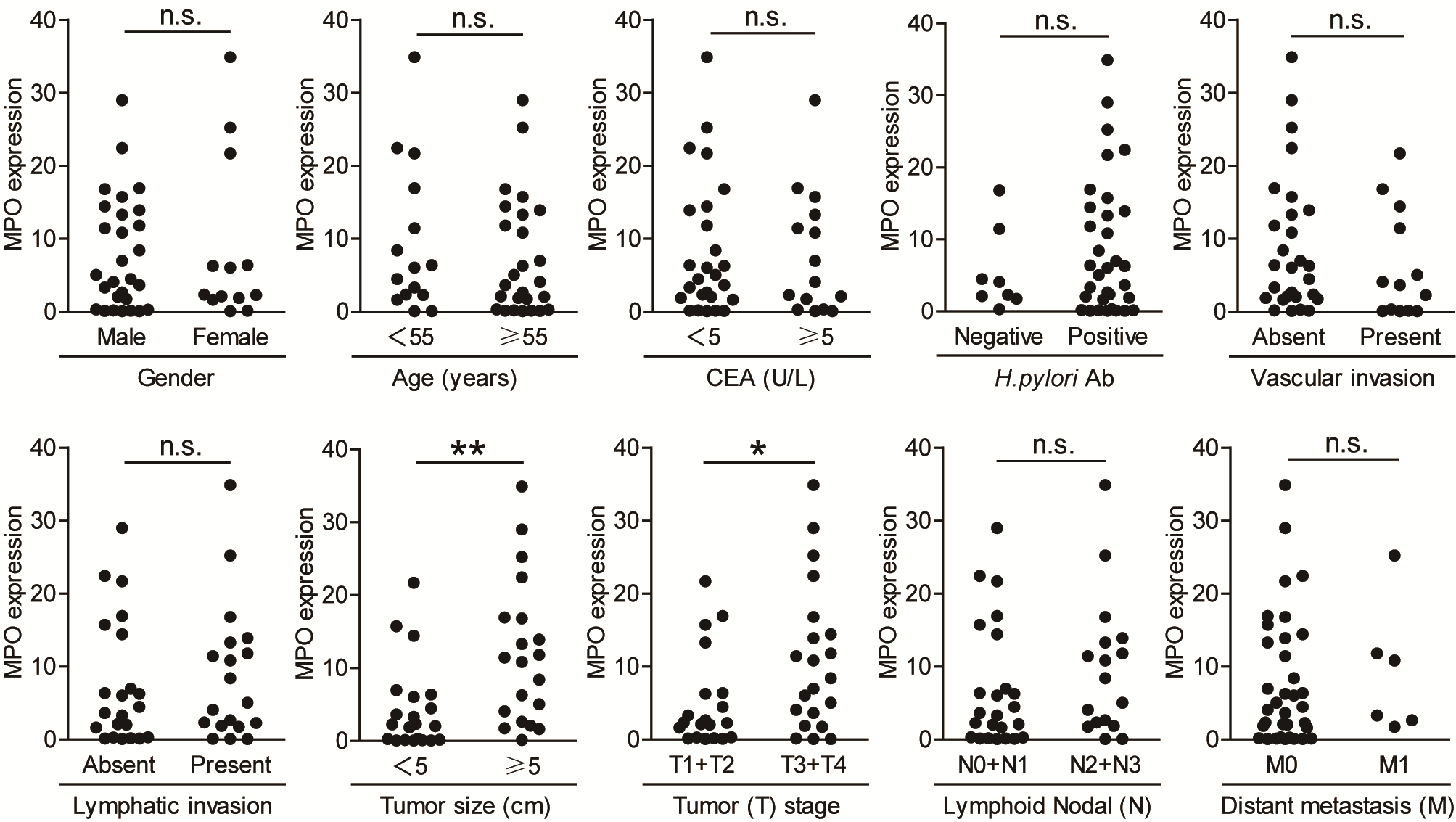


**Supplementary Figure 1.** MPO expression and its potential correlations with clinical parameters. MPO expression in GC tumors was analyzed for correlations with clinical pathological parameters. **P*<0.05, ***P*<0.01, n.s *P*>0.05 for groups connected by horizontal lines. Each dot represents 1 patient. CEA, carcinoembryonic antigen; *H. pylori* Ab, *Helicobacter pylori* antibody.

**
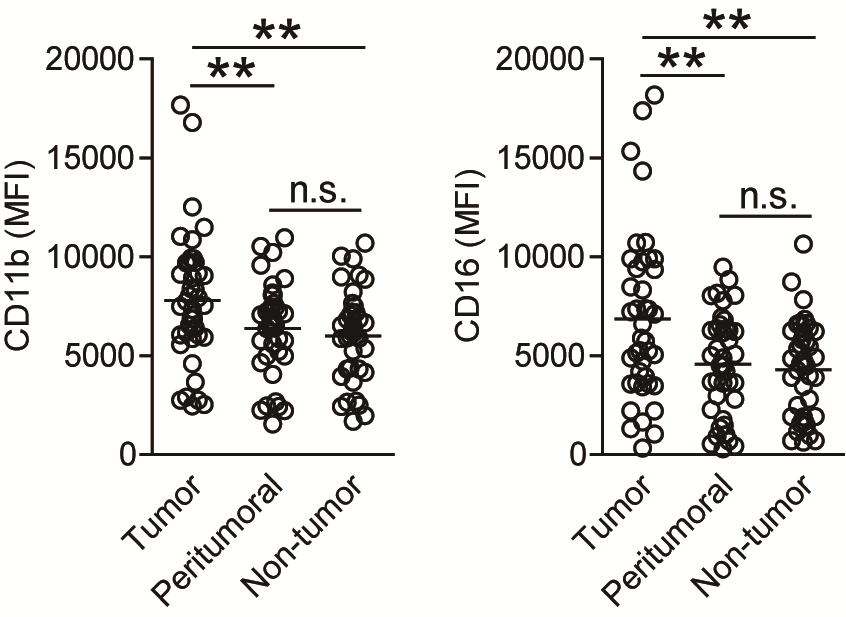
**

**Supplementary Figure 2.** Statistics analysis of expressions of CD11b and CD16 on neutrophils in each samples of patients with GC (n=41). The horizontal bars in panels represent mean values. Each ring in panels represents 1 patient. **P*<0.05, ***P*<0.01, n.s *P*>0.05 for groups connected by horizontal lines.

**
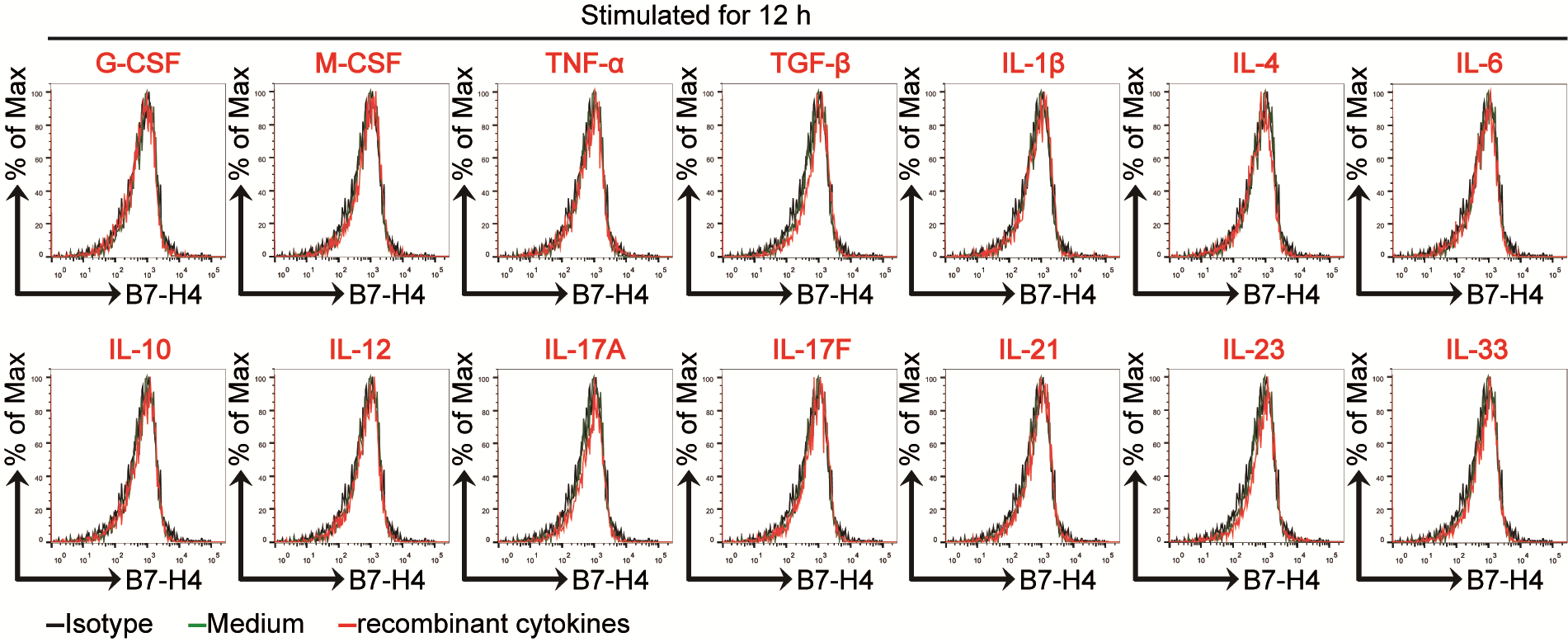
**

**Supplementary Figure 3.** Expression of B7-H4 on neutrophils exposed to G-CSF, M-CSF, TNF-α, TGF-β, IL-1β, IL-4, IL-6, IL-10, IL-12, IL-17A, IL-17F, IL-21, IL-23, or IL-33 (100 ng/ml) for 12 h. black, isotype control.

**
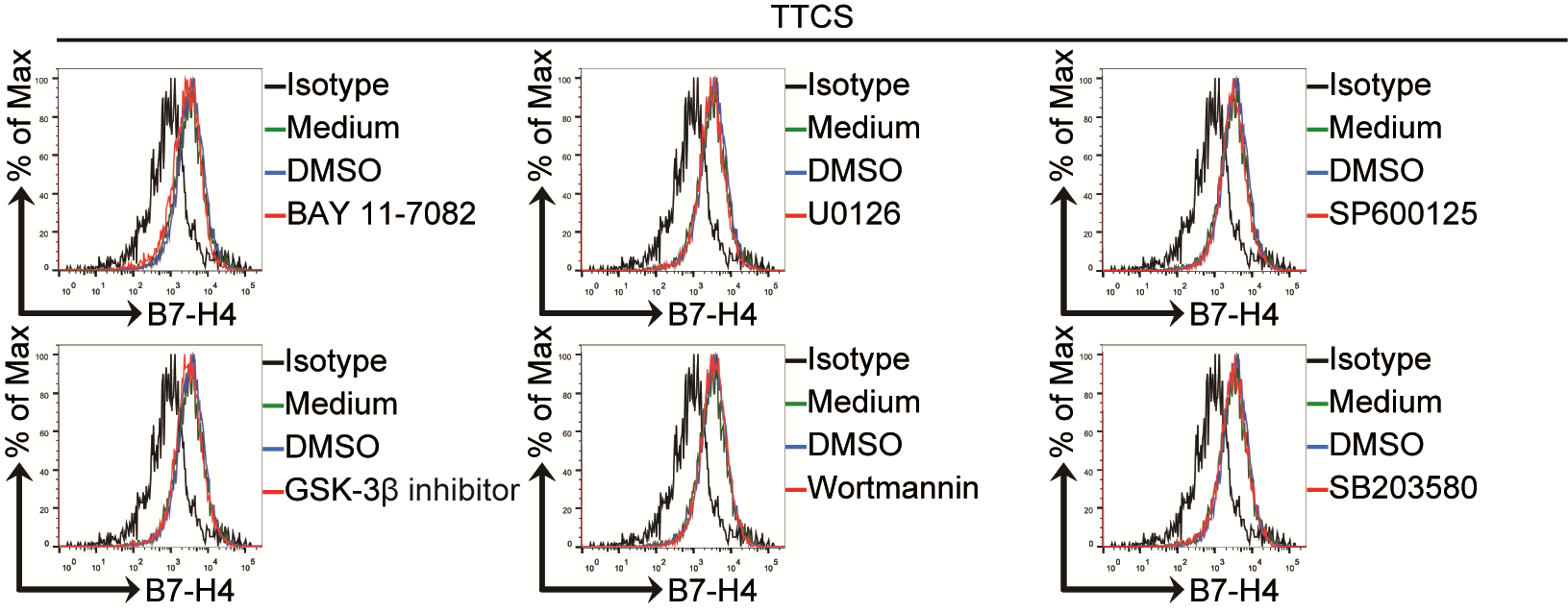
**

**Supplementary Figure 4.** Expression of B7-H4 on neutrophils exposed to 50% TTCS with or without BAY 11-7082 (an IκBα inhibitor), U0126 (an MEK-1 and MEK-2 inhibitor), SP600125 (a JNK inhibitor), SB203580 (a MAPK inhibitor), Wortmannin (a PI3K inhibitor), or GSK-3β inhibitor for 12 h. black, isotype control.

**
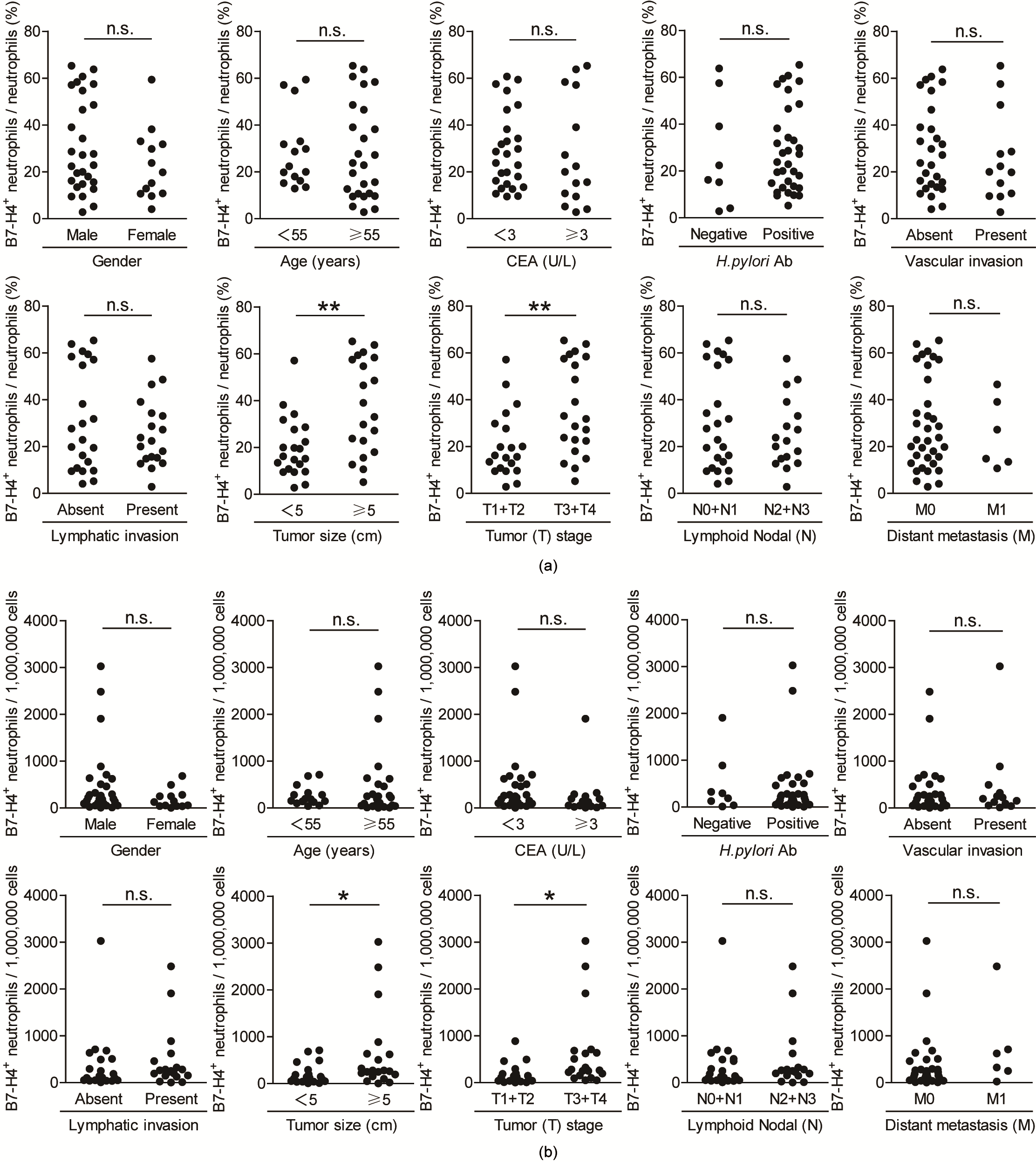
**

**Supplementary Figure 5.** Intratumoral B7-H4^+^ neutrophil percentage or number and its potential correlations with clinical parameters. Intratumoral B7-H4^+^ neutrophil percentage (a) or intratumoral B7-H4^+^ neutrophil number (b) in GC tumors was analyzed for correlations with clinical pathological parameters. **P*<0.05, ***P*<0.01, n.s *P*>0.05 for groups connected by horizontal lines. Each dot represents 1 patient. CEA, carcinoembryonic antigen; *H. pylori* Ab, *Helicobacter pylori* antibody.
