# Supplementary material for: Granulocyte-Macrophage Colony-Stimulating Factor-Activated Neutrophils Express B7-H4 That Correlates with Gastric Cancer Progression and Poor Patient Survival
Source: J Immunol Res. 2021 Mar 1;2021:6613247. doi: 10.1155/2021/6613247 (PMC7962878; doi:10.1155/2021/6613247)
Supplement: Supplementary 2 — Supplementary Table 1: antibodies and other reagents. Supplementary Table 2: clinical characteristics of 41 patients with gastric cancer. Supplementary Table 3: primer sequences for real-time PCR analysis. [file 6613247.f2.doc]

**Supplementary Table 1.** Antibodies and other reagents

| Antibodies and reagents | Manufacturers |
| --- | --- |
| Antibodies for flow cytometry |  |
| anti-CD45-PE-Cy7 | Biolegend |
| anti-CD11b-PerCP-Cy5.5 | Biolegend |
| anti-CD66b-FITC | Biolegend |
| anti-CD15-APC-Cy7 | Biolegend |
| anti-B7-H4-APC | Biolegend |
| anti-CD54-PE | Biolegend |
| anti-CD16-Brilliant™ Violet 605 | BD Biosciences |
| Antibodies for immunohistochemical staining |  |
| anti-MPO | Abcam |
| horseradish peroxidase anti-rabbit IgG | Zhongshan Biotechnology |
| Antibodies for neutralizing and blocking |  |
| anti-GM-CSF | R&D Systems |
| anti-GM-CSF receptor (GM-CSFR) | R&D Systems |
| Antibodies for western blot |  |
| anti-STAT3 | Abcam |
| anti-p-STAT3 (Y705)  anti-GAPDH | Abcam  Abcam |
| ELISA kits |  |
| GM-CSF | R&D Systems |
| Reagents for signaling pathways inhibition |  |
| STAT3 phosphorylation inhibitor FLLL32 | MedKoo Biosciences |
| JAK signaling inhibitor AG490  MEK-1 and MEK-2 inhibitor U0126 | Merk Millipore  Merk Millipore |
| IκBα inhibitor BAY 11-7082  GSK-3β inhibitor VI | Calbiochem  Calbiochem |
| JNK inhibitor SP600125 | Calbiochem |
| MAPK inhibitor SB203580 | Calbiochem |
| PI3K inhibitor Wortmannin | Calbiochem |
| Collagenase Ⅳ | Sigma-Aldrich |
| DNase Ⅰ | Sigma-Aldrich |
| DMSO | Sigma-Aldrich |
| Protein Extraction Reagent | Pierce |
| SuperSignal® West Dura Extended Duration Substrate kit | Thermo |
| Fetal calf serum (FCS) | Gibco |
| Penicillin/Streptomycin | Gibco |
| RPMI-1640 | Hyclone |
| Ficoll-Paque Plus | GE Healthcare |
| lyses solution | TIANGEN |
| TRIzol reagent | Invitrogen |
| PrimeScript^TM^ RT reagent Kit | TaKaRa |
| Real-time PCR Master Mix | Toyobo |
| All recombinant human cytokines | PeproTech |

APC-Cy7, allophycocyanin-cyanin 7; PE-Cy7, phycoerythrin-cyanin 7; FITC, Fluorescein isothiocyanate; PE, phycoerythrin; PerCP-Cy5.5, peridin chlorophyl protein-cyanin 5.5; APC, allophycocyanin.

**Supplementary Table 2.** Clinical characteristics of 41 patients with gastric cancer

| Variables | No. of patients |
| --- | --- |
| Gender (male/female) | 28/13 |
| Age (years; median, range) | 59, 24-75 |
| *H.pylori* Ab (negative/positive) | 8/33 |
| CEA (U/L; <3/≥3) | 26/15 |
| Tumor size (cm; <5/≥5) | 21/20 |
| Lymphatic invasion (absent/present) | 22/19 |
| Vascular invasion (absent/present) | 28/13 |
| Tumor (T) invasion (T1+T2/T3+T4) | 20/21 |
| Lymphoid Nodal (N) status (N0+N1/N2+N3) | 24/17 |
| Distant metastasis (M) status (M0/M1) | 35/6 |
| TNM stage (Ⅰ+Ⅱ/Ⅲ+Ⅳ) | 14/27 |
| MPO expression^a^ (median, range) | 4.486, 0.068-34.902 |

^a^MPO expression in tumor tissues was analyzed by real-time PCR. CEA, carcinoembryonic antigen; *H. pylori* Ab, *Helicobacter pylori* antibody; myeloperoxidase, MPO.

**Supplementary Table 3. Primer sequences for real-time PCR analysis**

| Gene | Primer | Sequence 5′→3′ |
| --- | --- | --- |
| Human MPO | forward | TGGTGGGAGAACGAGGGTGTG |
|  | reverse | CGGTGGTGATGCCTGTGTTGTC |
| Human GAPDH | forward | ACCCAGAAGACTGTGGATGG |
|  | reverse | CAGTGAGCTTCCCGTTCAG |
